# Anæsthesia, or the Employment of Chloroform and Ether, in Surgery, Midwifery, Etc.

**Published:** 1849-07

**Authors:** 


### ARTICLE III.

*Anæsthesia, or the employment of Chloroform and Ether in Surgery, Midwifery, etc.* By J. Y. Simpson, M.D., F.R.S.E., Professor of Midwifery in the University of Edinburgh, Physician-Accoucheur to the Queen in Scotland, etc., etc. Philadelphia: Lindsay & Blakiston. 1849. pp. 248 8vo. (From the Publishers—For sale by Griggs, Bross, & Co., Chicago.)

This is a work comprising a number of essays written at different times by Professor Simpson, upon the discovery, history, mode of operation, chemical properties, mode of administration, results from the use, etc., of the different anæsthetic agents.

The author believes fully in their utility, especially in midwifery and surgery, and contends that statistics prove that the rate of mortality is diminished under their use.

Those wishing to obtain a full knowledge of the subject and of the arguments in favor of anæsthesia, would do well to avail themselves of the information contained in these several papers from the pen of the distinguished Edinburgh Professor. H.

